# Experiments of three identical huts with shape-stabilized phase change materials and simulation of detached house in Japanese climate classification

**DOI:** 10.1016/j.dib.2018.09.095

**Published:** 2018-10-02

**Authors:** Hyun Bae Kim, Masayuki Mae, Youngjin Choi

**Affiliations:** Department of Architecture, Graduate School of Engineering, The University of Tokyo, Japan

## Abstract

The data in this article are the experiment and simulation results of three identical huts were examined using by using varying shape-stabilized PCMs (SSPCMs) sheet levels in winter of Chiba prefecture where Japanese temperate climate. A shape-stabilized phase-change material (SSPCM) established the melting- and solidification-temperature ranges at 19–26 °C was installed on the floor, walls, and ceiling of various buildings, and its effects on indoor room temperature stabilization and heating load reduction were examined using experiments and simulations. The PCM model was developed based on the specific heat capacity measured using a thermostatic chamber and simulations results were obtained using EnergyPlus. The validity of the PCM model was examined by comparing the simulation and experimental results. The model was then examined to determine the applicability of PCM to the various climates in Japan through annual heating load simulations. The target buildings were classified as Type A (no PCM, reference), Type B (only the floor contained PCM), and Type C (the floor, walls, and ceiling contained PCM) using a standard Japanese house. Types B and C had the same amount of PCM. The simulation was run for 21 cases, with one being run for each type of building in seven Japanese climates.

**Specifications table**

**Table:** 

Subject area	*Building and Environment*
More specific subject area	*Heating energy conservation, Heat load simulation*
Type of data	*Excel Files*
How data was acquired	*The data are measured by thermocouples in each hut, Energy plus simulation result by Japanese standard house.*
Data format	*.xls (Raw, analyzed)*
Experimental factors	*Comparison of indoor temperature and heating load according to PCM installation in Japanese climate classification*
Experimental features	*Average indoor temperature, surface temperature, heat flow on each surface, heating load*
Data source location	*Experiment location (Narita)*
	*Simulation city of Japan (Kitami, Iwamizawa, Morioka, Nagano, Utsunomiya, Okayama, Miyazaki)*
Data accessibility	*Data were obtained from the experiment and simulation accessible within this article*
Related research article	https://doi.org/10.1016/j.buildenv.2017.08.038

**Value of the data**

● Three identical huts using varying shape-stabilized PCMs (SSPCM) levels were examined indoor temperature.
● In Hut A, no SSPCM sheets were applied; in Hut B, four layers of SSPCM sheets were applied to the floor; in Hut C, one layer of SSPCM was applied to the floor, walls, and ceilings.
● The simulation was examined to determine the applicability of PCM to the various climates in Japan.
● The target buildings were classified as Type A (no PCM, reference), Type B (only the floor contained PCM), and Type C (the floor, walls, and ceiling contained PCM) using a standard Japanese house (Types B and C had the same amount of PCM).
● The simulation was run for 21 cases, with one being run for each type of building in seven Japanese climates.

## 1 Data

**Fig. 1:**
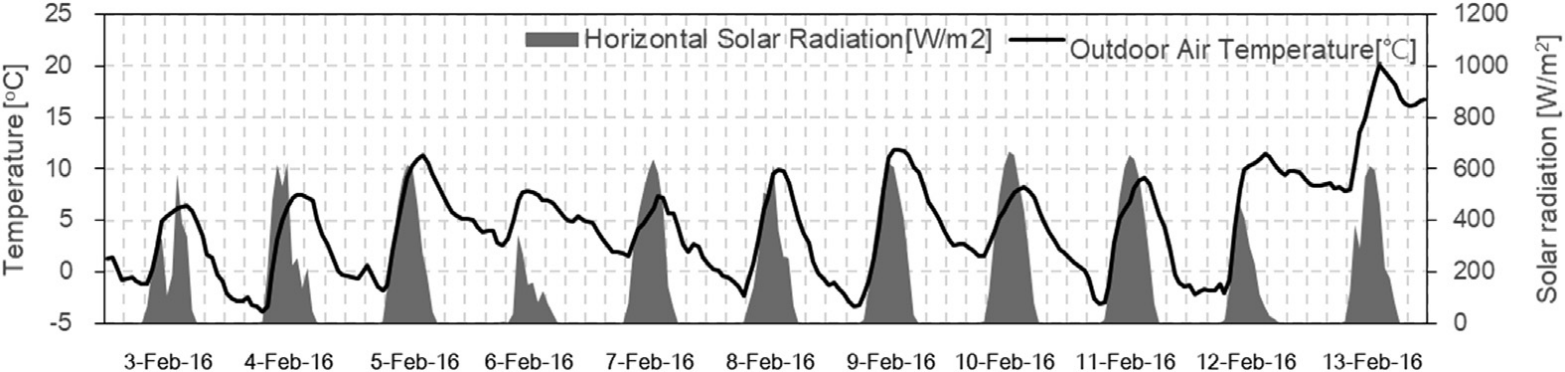
Outdoor temperature and horizontal solar radiation during January 3–13 (experiment).

**Fig. 2:**
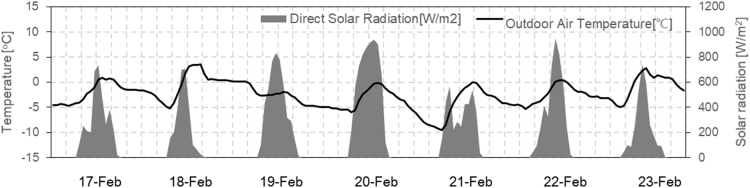
Outdoor temperature and direct solar radiation during January 17–23 (simulation).

This article data set is show of comprised of indoor temperature according to SSPCM sheets (paraffin based PCM mixed with polypropylene and elastomer) installation [1]. The results of indoor temperature in each hut during experiments were evaluated during February 3rd–13th. A weather station that measures outdoor temperature and pyrheliometers were measured horizontal solar radiation as shown in Fig. 1. The data sheets show the specific heat and enthalpy of SSPCM sheet measured by thermostatic chamber and simulation validation result. Annual heating load was calculated as 21 cases, with one being run for each type of building in seven Japanese climates. The data sheets show the result of indoor temperatures, sensible heating energy, surface temperature and heat flow for the winter heating period from February 17th to 23th in the living room on the first floor in climate zone 3 (Morioka, HDD:3207). The weather condition during the simulation as Fig. 2.

## 2 Experimental design, materials and methods

In November 2014, three identical lightweight test huts were constructed in the Chiba Prefecture, Japan; the building and window specifications are explained in original: Hut A was used as a reference and contained no PCM, Hut B was equipped with four SSPCM sheet layers on its floor, and Hut C had one SSPCM sheet layer each installed on the floor, walls, and ceiling (Huts B and C had the same amount of PCM). The thermocouples installed at different heights (100 mm, 600 mm, 1100 mm, and 1700 mm) at five points (east, west, north, south, and center) of the floor of each hut in order to measure the air temperature within the hut [1]. PCM model made from the specific heat capacities of the SSPCM sheets, which were acquired from the thermostatic chamber experiment. The validity of PCM model was examined by comparing the simulation results with the experimental results. Annual heating load were run for twenty-one cases, with one being run for each type (none: A, only the floor: B, all surface contained PCM: C, B and C had the same amount of PCM) using a standard Japanese house according to the energy saving standard in seven Japanese climates [2], [3].
